# High expression of ANRIL correlated with the poor prognosis in patients with cancer: A meta-analysis

**DOI:** 10.1097/MD.0000000000030531

**Published:** 2022-09-09

**Authors:** Yun Liu, Linqi Zhu, Wenjun Zhao, Yong Zhou, Shihe Shao

**Affiliations:** a Department of Digestive, The Affiliated People’s Hospital, Jiangsu University, Zhenjiang, Jiangsu, China; b School of Medicine, Jiangsu University, Zhenjiang, Jiangsu, China.

**Keywords:** ANRIL, cancer, meta-analysis, poor prognosis

## Abstract

ANRIL, also called CDKN2B antisense RNA 1, is an important genetic susceptibility locus for cardiovascular diseases and associated with numerous pathologies, including several human cancers.

**Objective:**

The relationship between ANRIL and the clinical outcome or prognosis of cancer patients was analyzed in this meta-analysis.

**Methods:**

One thousand seven hundred eight cancer patients were selected in 23 studies from 3 databases (Pubmed, Cochrane Library, and EMBASE).

**Results:**

A fixed-effects model indicated that the high expression of ANRIL is obviously linked to poor overall survival (OS) (Hazard ratio [HR] = 1.77, 95% confidence interval [CI] = 1.57–2.00, *P* < .00001); the random-effects model revealed poor disease-free survival (DFS) (HR = 1.86, 95% CI: 1.46–2.37, *P* < .00001). A high level of ANRIL expression was also associated with the tumor size (small vs large, odds ratio [OR] = 0.57, 95% CI: 0.39–0.83, *P* = .003), TNM stage (I + II vs III + IV; OR = 0.40, 95% CI: 0.24–0.69, *P* = .0008), and lymph node metastasis (LNM) (Yes vs No, OR = 3.66, 95% CI: 1.46–9.17, *P* = .006). ANRIL was not related significantly to histologic differentiation compared to poor with moderate + well; the OR value is 0.74, 95% CI: 0.26–2.12, *P* = .58. In addition, evidence suggested that a high level of ANRIL was positively associated with human cancer type, follow-up time, and sample size.

**Conclusion:**

This meta-analysis demonstrated that ANRIL may be a valuable biomarker for predicting poor prognosis in cancer patients.

## 1. Introduction

Long non-coding RNAs (lncRNAs) are defined as transcripts > 200 nt in length that cannot code proteins.^[1,2]^ In recent years, more and more studies have focused on lncRNAs and found them to be involved in the regulation of various intracellular processes; but the type, quantity, and function are unclear. Several studies found that lncRNAs were correlated closely with cancers and that a high level of ANRIL expression is closely associated with a poor prognosis, overall survival (OS), and disease-free survival (DFS) in various human cancers.^[3–6]^ Furthermore, a high ANRIL levels was related to the metastasis and proliferation of tumor cells.

A meta-analysis on ANRIL with human cancer has been reported, but one focused on lymph node metastasis (LNM) and prognosis in human cancer,^[7]^ in another in 2016 included only 6 studies.^[8]^ With the increase in lncRNA research, many studies of ANRIL in human cancer have been reported. This meta-analysis investigated the relationship between ANRIL and the clinicopathological parameters of human cancer patients detected in some studies.

## 2. Materials and methods

### 2.1. Literature search strategies

Three databases (Pubmed, Cochrane Library, and EMBASE) were used to search the articles related to “ANRIL” or “ANRIL long non-coding RNA” “CDKN2B-AS long non-coding RNA” or “antisense noncoding RNA in the INK4 locus” “CDKN2B antisense RNA” or “ANRIL lncRNA” or “ANRIL long ncRNA” and “cancer(s)” or “tumor(s)” or “tumour(s)” or “Neoplas*” and “prognos*”or “surviv*” or “outcome” or “mortality” or “predict”. A list of references was retrieved manually to obtain relevant articles and review papers to identify potential studies. The literature was published up to November 30, 2019.

### 2.2. Inclusion and exclusion criteria

The inclusion criteria were as follows: Studies were written in English; articles exploring the relationship between high and low expression of ANRIL and human cancer prognosis; articles containing the clinicopathologic parameters or clinical data including OS or DFS, tumor size, LNM, TNM stage, and histologic differentiation; the inclusion of sufficient data for the calculating hazard ratio (HR) and its 95% confidence intervals (CI). The exclusion criteria were as follows: duplicate publications; review articles, meta-analyses, case reports, and non-human researches; studies that lacked relevant data or whose data cannot be calculated its HR, 95% CI, and *P* values.

### 2.3. Data extraction and study quality assessment

The first author’s name, publication year, country of origin, sample size, sample type, cancer type, the number of patients, tumor size, LNM, TNM stage, histologic differentiation and cut-off value, and follow-up time were extracted from eligible studies. Furthermore, the HR and corresponding 95% CI were acquired from the article directly or calculated independently from the outcome (OS and DFS) using the Engauge Digitizer 4.1 (http://digitizer.sourceforge.net/).^[8]^

The Newcastle-Ottawa quality assessment scale was used to evaluate the study quality. The Newcastle-Ottawa quality assessment scale score items included the outcome, selection, and comparability ranging between 0 and 9.

### 2.4. Statistical analysis

Stata statistical software version 12.0 (Stata Corporation, College Station, TX) and RevMan5.3 software (Cochrane Collaboration, http://www.cc-ims.net/RevMan/relnotes.htm/) were used to analyze the data extracted from this meta-analysis. The heterogeneity of the eligible studies was detected by *I*^2^ statistics and a chi-square *Q* test. The random-effects and fixed-effects models were chosen according to the difference in heterogeneity.^[9]^ Funnel plot, Begg test, and Egger test were applied to evaluate the potential publication bias. Sensitivity analysis was performed to assess whether the individual studies affect the overall results.

## 3. Results

### 3.1. Literature search and characteristics of eligible studies

**Figure 1.**
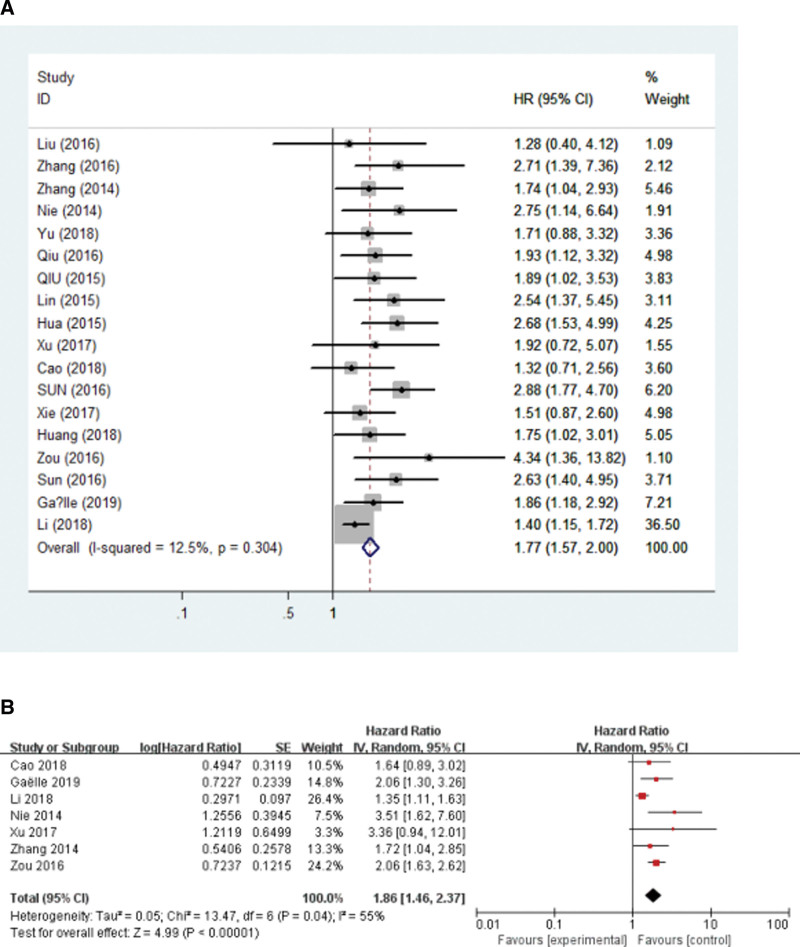
Forest plot for the relationship between the expression level of ANRIL with OS and DFS. (A) OS, (B) DFS. DFS = disease-free survival, OS = overall survival.

As shown in Figure 1, 169 studies were selected in the initial screening, but 54 duplicate studies were excluded. Then titles and abstracts were then read carefully, resulting in the exclusion of 84 irrelevant articles, leaving 31 potentially eligible studies. Finally, the whole papers were screened in full; 8 studies were excluded because of a lack of survival outcomes or clinicopathological parameters. Hence, 23 articles were finally selected for this meta-analysis.^[3–6,11–29]^

Research on lncRNAs has emerged in recent years. All 23 articles associated with ANRIL lncRNA published from 2014 to 2019 were written in English. Of these, 19 studies were from China, 2 from Iran, and only 1 from France, and 1 from the Czech Republic; 1708 cancer patients were included. Table 1 lists the main characteristics. Of the 23 studies, 2 focused on gastric cancer, 2 focused on hepatocellular carcinoma, 1 on gallbladder cancer, 1 on intrahepatic cholangiocarcinoma, 1 on nasopharyngeal carcinoma, 1 on esophageal squamous cell carcinoma, 3 on non-small cell lung cancer, 1 on serous ovarian cancer, 1 on epithelial ovarian cancer, 1 on ovarian cancer, 3 on colorectal cancer, 1 on cervical cancer, 1 on lung adenocarcinoma, 1 on triple-negative breast cancer, 1 on osteosarcoma (OS), 1 on breast cancer, and 1 on perihilar cholangiocarcinoma. Of the 23 studies, 22 studies collected tissue and 1 collected serum. All used quantitative real-time PCR to detect the expression of ANRIL.

**Table 1:** Characteristics of the studies included in this meta-analysis.

Author	Year	Country	Sample size	Sample type	Cancer type	TNM stage	LNM	Follow up	Cut-off value	Outcome	HR statistics	NOS
						I/II Vs. III/IV	Yes versus No	Month				
Liu	2016	China	84	Tissue	GBC	16/68	58/26	60	Median value	OS	Survival curve	7
Zhang	2016	China	53	Tissue	CEC	27/26	13/40	60	Median value	OS	Reported	8
Zhang	2014	China	120	Tissue	GC	66/54	71/49	60	fold change ^≥^ 3	OS/DFS	Reported	8
Nie	2014	China	68	Tissue	NSCLC	45/23	38/30	40	Median value	OS/DFS	Reported	8
Gaëlle	2019	France	39	Tissue	iCCA	NA	11/28	120	NA	OS/DFS	Survival curve	7
Yu	2018	China	57	Tissue	OS	NA	25/32	60	Expression level	OS	Survival curve	7
Qiu	2016	China	102	Tissue	EOC	33/69	NA	80	Median value	OS	Reported	7
QIU	2015	China	68	Tissue	SOC	19/49	40/28	80	Median value	OS	Reported	8
Lin	2015	China	87	Tissue	NSCLC	26/61	47/40	60	Median value	OS	Reported	8
Hua	2015	China	92	Tissue	HCC	39/53	NA	60	Median value	OS	Reported	8
Mostafa	2015	Iran	38	Tissue	BC	22/16	12/26	NA	Expression level	NA	NA	7
Xu	2017	China	50	Tissue	LAD	33/17	NA	NA	NA	DFS	Reported	8
Roghayeh	2019	Iran	39	Tissue	GC	15/24	NA	NA	Expression level	NA	NA	7
Xu	2017	China	37	Tissue	TNBC	23/14	15/22	60	Median value	OS	Survival curve	7
Cao	2018	China	50	Tissue	ESCC	18/32	37/13	60	Median value	OS/DFS	Reported	7
SUN	2016	China	97	Tissue	CRC	33/64	NA	60	Expression level	OS	Survival curve	7
Xie	2018	China	100	Serum	NSCLC	70/30	48/52	72	NA	OS	Reported	7
Huang	2018	China	100	Tissue	HCC	80/20	NA	60	Median value	OS	Survival curve	7
Zou	2016	China	88	Tissue	NPC	50/38	NA	80	Median value	OS/DFS	Reported	7
Sun	2016	China	108	Tissue	CRC	59/49	58/50	60	Median value	OS	Reported	8
Li	2018	China	82	Tissue	PHCC	41/41	NA	60	Median value	OS/DFS	Reported	7
Thiele	2018	Czech	63	Tissue	CRC	44/11	NA	55	NA	NA	NA	7
Miao	2019	China	86	Tissue	Ovarian	35/51	NA	NA	NA	NA	NA	7

BC = breast cancer, CEC = cervical cancer, CRC = colorectal cancer, DFS = disease-free survival, EOC = epithelial ovarian cancer, ESCC = esophageal squamous cell carcinoma, GBC = gallbladder cancer, GC = gastric cancer, HCC = hepatocellular carcinoma, iCCA = interahepatic cholangiocarcinoma, LAD = lung adenocarcinoma, NPC = nasopharyngeal carcinoma, NSCLC = non-small cell lung cancer, OS = osteosarcoma, PHCC = perihilar cholangiocarcinoma, SOC = serous ovarian cancer, TNBC = triple-negative breast cancer.

### 3.2. Association between high ANRIL and OS in human cancer

Of the 23 studies, 18 suggested OS according to the ANRIL expression levels in 1432 patients. As shown in Figure 1A, high ANRIL has a significant association with poor OS in cancer patients. The pooled HR was 1.77 (95% CI = 1.57–2.00, *P* < .001). *I*^2^ = 12.5%, no obvious heterogeneity, and the fixed-effects model was chosen. In Figure S1, Supplemental Digital Content 1, http://links.lww.com/MD/H300, when Li 2018 was excluded, the value of *I*^2^ decreased to 0%. In addition, the results showed that high levels of ANRIL expression were a predictor of a poor prognosis among human cancers.

Although there was no obvious heterogeneity in it, subgroup analysis was performed using a fixed-effects model on the cancer type, follow-up time, and sample size. According to the result, high expression of ANRIL is associated with a poor prognosis of digestive system tumors (HR = 1.69, 95% CI: 1.46–1.95, *P* < .0001) and other tumors (HR = 2.03, 95% CI: 1.61–2.57, *P* < .0001). Meanwhile, there was also a significant correlation between the high level of ANRIL expression and the OS of patients with cancer with a follow-up time equal to or <5 years (HR = 1.75, 95% CI: 1.52–2.01, *P* < .0001) and >5 years (HR = 1.87, 95% CI: 1.44–2.42, *P* < .0001). In addition, a high level of ANRIL expression was associated closely with patients with a poor OS in sample sizes <100 (HR = 1.75, 95% CI: 1.53–2.02, *P* < .0001) and equal to or >100 (HR = 1.84, 95% CI: 1.44–2.36, *P* < .0001) (Table 2).

**Table 2:** Results of subgroup analysis of OS with pooled HRs in patients with overexpression of ANRIL.

Outcome	Studies (n)	HR	95% CI	*P* value	Model	Heterogeneity	
						*I*^2^ (%)	*P* value
Cancer type							
Digestive system	9	1.69	1.46–1.95	<.0001	Fixed	39.3	.106
Other tumor	9	2.03	1.61–2.57	<.0001	Fixed	0.0	.812
Follow-up time							
≤5	13	1.75	1.52–2.01	<.0001	Fixed	27.7	.166
>5	5	1.87	1.44–2.42	<.0001	Fixed	0.0	.619
Sample size							
<100	13	1.75	1.53–2.02	<.0001	Fixed	31.3	.132
≥100	5	1.84	1.44–2.36	<.0001	Fixed	0.0	.764

CI = confidence interval, HR = hazard ratio, OS = osteosarcoma.

### 3.3. Association between ANRIL and DFS

Seven studies containing 497 patients were selected to analyze the association between the ANRIL level and DFS. The random-effects model was used because of the higher heterogeneity (*I*^2^ = 55%, *P* = .04) in the studies. The results demonstrated that the expression level of ANRIL was related to DFS (HR = 1.86, 95% CI: 1.46–2.37, *P* < .00001) (Fig. 1B). This suggests that a high level of ANRIL correlated with a poor DFS in human cancers.

### 3.4. Correlation of ANRIL expression with clinicopathological parameters

**Figure 2.**
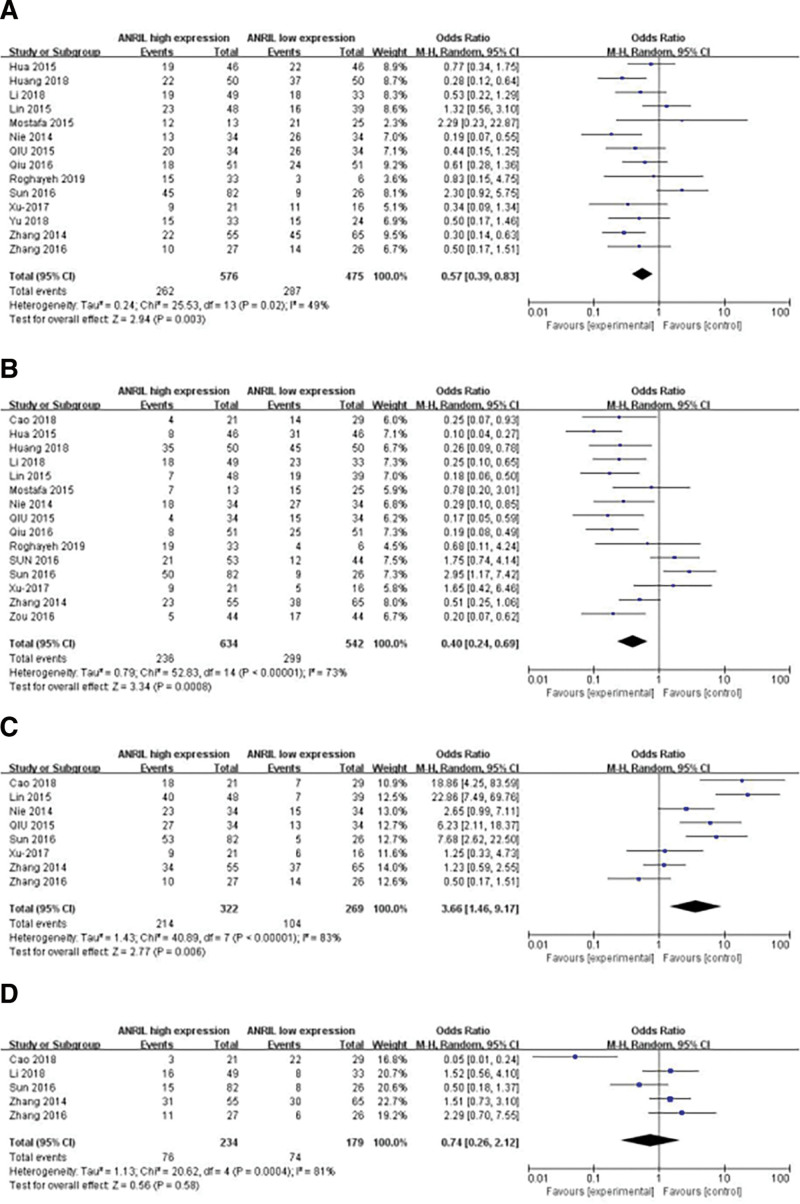
Forest plot of the expression of ANRIL and OR for clinicopathological parameters. The researched clinicopathological parameters are (A) tumor size, (B) TNM stage, (C) LNM, (D) histologic differentiation. OR = odds ratio, LNM = lymph node metastasis.

The random-effects model was performed to analyze the relationship between high expression of ANRIL and LNM, TNM stage, and histologic differentiation. The results shown that the ANRIL level correlated with the tumor size (small vs larger; odds ratio [OR] = 0.57, 95% CI: 0.39–0.83, *P* = .003; Fig. 2A). Fifteen studies reported the expression of ANRIL connected with the TNM stage (I + II vs III + IV; OR = 0.40, 95% CI: 0.24–0.69, *P* = .0008; Fig. 2B). Eight articles reported a high level of ANRIL expression associated with LNM in about a pool of 591 patients (OR = 3.66, 95% CI: 1.46–9.17, *P* = .006; Fig. 2C). Furthermore, the expression of ANRIL was not related to histologic differentiation (poor vs well + moderate, OR = 0.74, 95% CI: 0.26–2.12, *P* = .58; Fig. 2D).

### 3.5. Publication bias

**Figure 3.**
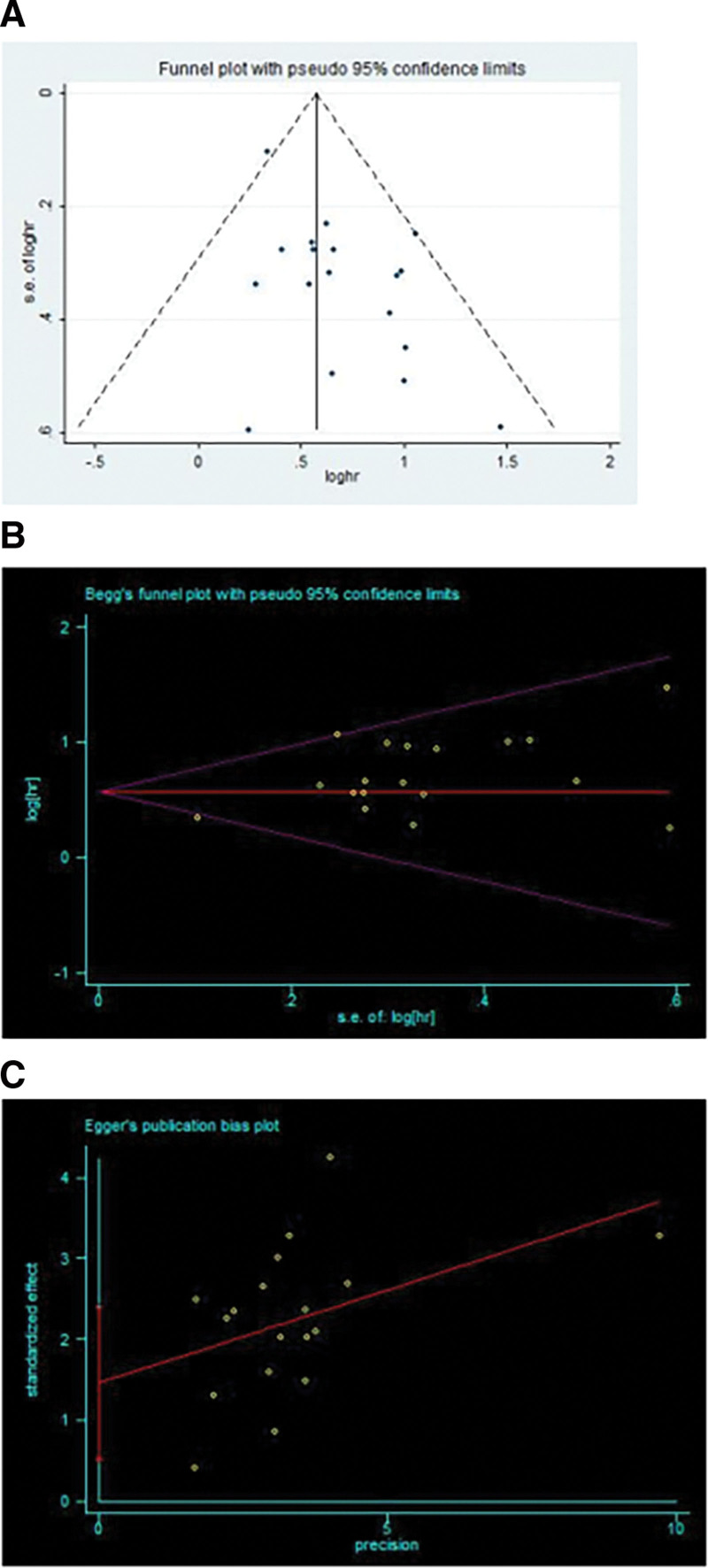
Evaluating the publication bias on OS (A) Funnel plot, (B) Begg funnel plot, (C) Egger publication bias plot. OS = overall survival.

In this meta-analysis, the funnel plot, Begg test, and Egger test were performed to analyze the publication bias. There is a certain extent of asymmetry in the form of the funnel plot (Fig. 3A). The Begg test was used to detect the publication bias in which it was found that *P* = .596 > 0.05 (Fig. 3B), while the Egger test showed that *P* = .005 < 0.05 (Fig. 3C). Hence, the 2 methods did not reach the same conclusion. Therefore, it could be concluded that there was a publication bias in these studies.

### 3.6. ANRIL expression in different cancer types

**Figure 4.**
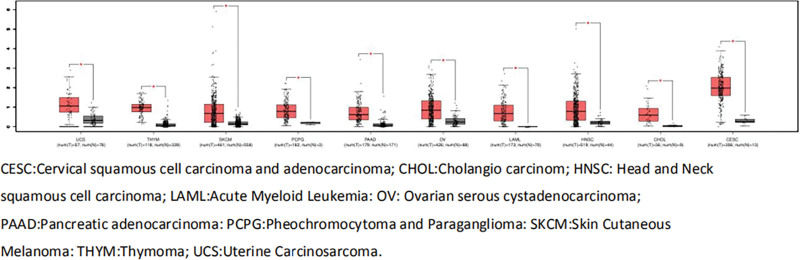
The expression of ANRIL in different tumor tissues and the corresponding normal tissues.

The mRNA level of ANRIL was analyzed further in different cancer types. A newly interactive web server GEPIA that can be used to analyze the RNA-seq data from the TCGA and the GTEx projects was used to maintain the level of ANRIL expression in other cancer types. As shown in Figure 4, the level of ANRIL appeared higher in human tumor tissues than in the corresponding normal tissues.

### 3.7. Sensitivity analysis

**Figure 5.**
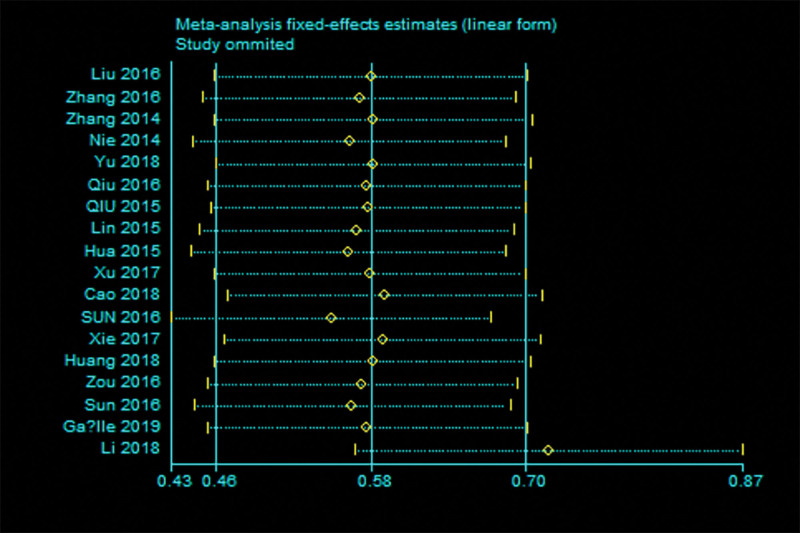
The sensitivity analysis was used to analyze the effect of the individual studies on the pooled HRs for the relationship between ANRIL expression and OS. HR = hazard ration, OS = overall survival.

Sensitivity analysis was used to evaluate the effects of a single study on the overall meta-analysis by deleting an eligible study at a time from the pooled analysis. The results showed that this meta-analysis, inspired by Li 2018 (Fig. 5), is somewhat robust. When the Li 2018 was excluded, the exclusion of each study had no influence (Figure S2, Supplemental Digital Content 2, http://links.lww.com/MD/H301). Furthermore, after deletion, the result of the synthetic analysis has no heterogeneity but was more robust (Figure S3, Supplemental Digital Content 3, http://links.lww.com/MD/H302). All the supplementary data suggested that the synthetic analysis was robust after deleting Li 2018.

## 4. Discussion

According to some studies, ANRIL, a lncRNA, is increased in various malignancies, such as lung, liver, stomach, breast, and colon cancer.^[3,6,14,16,18]^ As a new oncogene, it regulates the proliferation, autophagy, metastasis, and other biological behaviors of tumor cells and is involved in the occurrence and development of tumors. In addition, it can reduce the survival time of cancer patients.

Pasmant et al. first identified ANRIL was in a familial melanoma neural system tumor in 2007.^[20,31]^ Increasing evidence has shown that the high ANRIL expression promotes biological behaviors, such as proliferation, migration, invasion and facilitates the epithelial-mesenchymal transformation process, but it suppresses cell apoptosis to a certain degree.^[18–21]^ ANRIL can regulate microRNA in human cancers, such as the regulation of nasopharyngeal carcinoma, colorectal cancer, prostate cancer, and ovarian cancer by let-7a,^[29,30]^ and retinoblastoma cells and triple-negative breast cancer by miR-99a.^[16]^ On the other hand, some studies have shown that ANRIL could promote cancer development and a poor prognosis through the PI3K signal pathway. For example, Guangyang Yu et al reported that high expression of ANRIL connected with the development and prognosis of osteosarcoma and regulated the function of OS cells by the AKT signal pathway.^[13]^ Dongli Zhang et al reported that the PI3K/Akt signal pathway could be inactivated by inhibiting ANRIL in cervical cancer cells.^[15]^ In retinoblastoma Y79 cells and retinal pigment epithelial ARPE-19 cells, high levels of ANRIL expression promoted migration and invasion through activating MEK/ERK and Wnt/β-catenin signal pathway can also downregulate miR-24.^[32]^

This meta-analysis aimed to explore the association between ANRIL and the clinical outcomes in human cancers. The data suggested that high ANRIL levels were correlated closely with poor OS in cancer patients. In addition, it is also closely related to a poor DFS. ANRIL expression was higher in tumor tissues than in the corresponding normal tissues.

Subgroup analysis showed that the expression of ANRIL was significantly related to the cancer type, follow-up time, and sample sizes. Furthermore, high ANRIL levels were significantly associated with the tumor size, LNM, and TNM stage but had no significant association with poor histologic differentiation. In addition, Li 2018 discovered that high ANRIL levels predict poor prognosis and promote metastasis and proliferation in perihilar cholangiocarcinoma. It was also the main origin of heterogeneity and instability in this meta-analysis. The existing heterogeneity was decreased when Li 2018 was excluded; the remaining 17 studies found a pooled HR of 0.71 (95% CI: 0.56–0.86, *P* < .0001) for high ANRIL expression, compared to the low ANRIL expression group (*I*^2^ = 0.0%, *P* = .807). Moreover, there was no obvious asymmetry in the shape of the funnel plot. The Begg test and Egger test revealed *P* = .592 > 0.05 and *P* = .476 > 0.05, respectively, which have consistent results and no significance, it is concluded that excluding Li 2018 could weaken the publication bias.

Nevertheless, several shortcomings should not be ignored in this meta-analysis. First, almost all articles were produced by Chinese researchers. In addition, 6 studies did not report the HR but provided the OS. The values of HR and 95% CI were obtained using Engauge Digitizer 4.1. The value of 1 study was from another meta-analysis.^[8]^ Overall, notwithstanding some limits in this article, it provided obvious evidence that high levels of ANRIL expression are linked with the tumor size, LNM, TNM stage, and poor OS and DFS in different human cancers but had no relationship with histologic differentiation. Moreover, ANRIL might be a valid biomarker for predicting poor prognosis in human cancers. Therefore, the findings of this meta-analysis with a preference for smaller studies and data will need to be confirmed in future studies.

## Author contributions

**Data curation:** Yun Liu, Linqi Zhu, Yong Zhou.

**Formal analysis:** Linqi Zhu.

**Funding acquisition:** Yun Liu, Shihe Shao.

**Methodology:** Yun Liu.

**Resources:** Linqi Zhu.

**Software:** Yun Liu, Wenjun Zhao, Yong Zhou.

**Validation:** Shihe Shao.

**Writing – original draft:** Yun Liu.

**Writing – review & editing:** Yong Zhou, Shihe Shao.

## Supplementary Material


