# Supplementary material for: High expression of ANRIL correlated with the poor prognosis in patients with cancer: A meta-analysis
Source: Medicine (Baltimore). 2022 Sep 9;101(36):e30531. doi: 10.1097/MD.0000000000030531 (PMC10980395; doi:10.1097/MD.0000000000030531)

Supplementary figure 1: Forest plot of HR for ANRIL high expression and overall survival when deleted Li 2018.

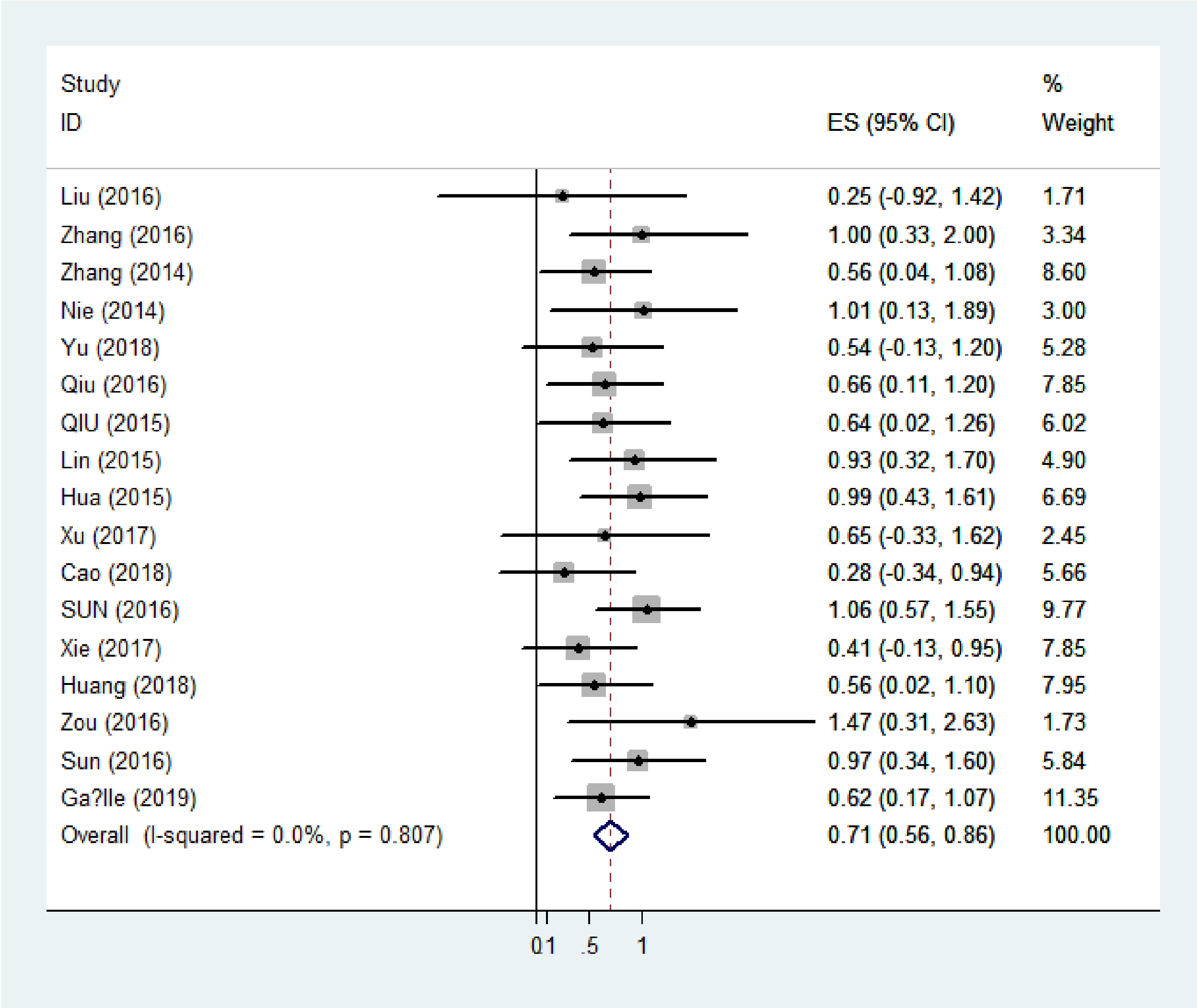

Supplement: Supplementary file 1 [file medi-101-e30531-s001.pdf]
