# Supplementary material for: High expression of ANRIL correlated with the poor prognosis in patients with cancer: A meta-analysis
Source: Medicine (Baltimore). 2022 Sep 9;101(36):e30531. doi: 10.1097/MD.0000000000030531 (PMC10980395; doi:10.1097/MD.0000000000030531)

Supplementary figure 2: Sensitivity analysis of the effect of the individual study on the pooled HRs for the correlation between ANRIL expression and OS when deleted Li 2018.

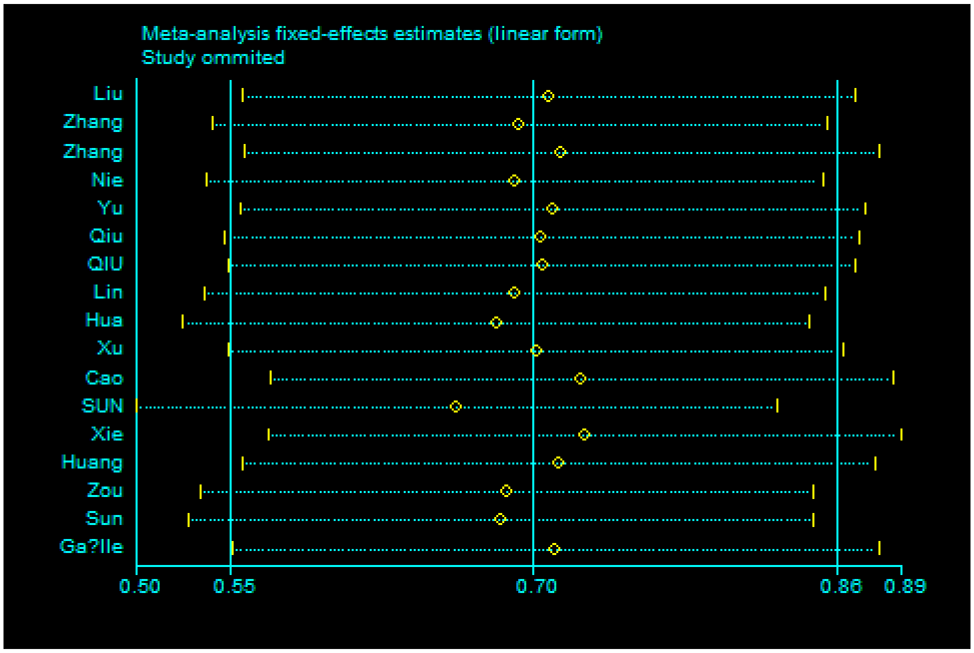

Supplement: Supplementary file 2 [file medi-101-e30531-s002.pdf]
